# Physical Extraction and Feature Fusion for Multi-Mode Signals in a Measurement System for Patients in Rehabilitation Exoskeleton

**DOI:** 10.3390/s18082588

**Published:** 2018-08-07

**Authors:** Canjun Yang, Qianxiao Wei, Xin Wu, Zhangyi Ma, Qiaoling Chen, Xin Wang, Hansong Wang, Wu Fan

**Affiliations:** State Key Laboratory of Fluid Power and Mechatronic Systems, Zhejiang University, Hangzhou 310027, China; wuhsin@zju.edu.cn (X.W.); mazhangyi2018@sina.com (Z.M.); qiaolingchen1@gmail.com (Q.C.); wangxin96_2015@163.com (X.W.); zjuwhs@zju.edu.cn (H.W.); zjufanwu@zju.edu.cn (W.F.)

**Keywords:** inertial measurement unit, visual measurement unit, data fusion, exoskeleton robot, Kalman filter

## Abstract

Measurement system of exoskeleton robots can reflect the state of the patient. In this study, we combined an inertial measurement unit and a visual measurement unit to obtain a repeatable fusion measurement system to compensate for the deficiencies of the single data acquisition mode used by exoskeletons. Inertial measurement unit is comprised four distributed angle sensors. Triaxial acceleration and angular velocity information were transmitted to an upper computer by Bluetooth. The data sent to the control center were processed by a Kalman filter to eliminate any noise. Visual measurement unit uses camera to acquire real time images and related data information. The two data acquisition methods were fused and have its weight. Comparisons of the fusion results with individual measurement results demonstrated that the data fusion method could effectively improve the accuracy of system. It provides a set of accurate real-time measurements for patients in rehabilitation exoskeleton and data support for effective control of exoskeleton robot.

## 1. Introduction

Rehabilitation exoskeletons robot are emerging as important components of the rehabilitation training process for patients affected by hemiplegia and cerebral apoplexy [1,2]. The rehabilitation training system comprises a lower extremity exoskeleton, where the robotics are employed for sensing, control, information fusion and mobile computing [3,4,5,6]. Accurate data from measurement systems can be provided to the feedback control of exoskeleton system. By combining human intelligence with the physical exoskeleton, the robotic system can complete tasks via a man-machine interaction. Therefore, the methods used in measurement systems for obtaining lower limb gesture is of great significance.

Worldwide famous exoskeleton robots have been equipped with related measurement system. The measurement system in the ReWalk exoskeleton [7] developed by an Israeli researcher sends grit data obtained from a gyroscope sensor to a data processing center. The HAL exoskeleton developed at Tsukuba University in Japan, the measurement system is based on acquiring and analyzing EMG signals [8] as well as plantar pressure signals from the wearer, through dividing the gait it can control each phase while walking. Ekso [9], developed by an American company, uses crutches with attached sensors as well as plantar pressure shoes and an upper limb accelerometer to detect the walking intentions of the wearer. However, these detection systems focus only on a single mode during information acquisition and their accuracy is difficult to verify. In addition, the information obtained by these sensor systems exhibits hysteresis. Previous studies have shown that the attitude error increases as objects move, which can be eliminated by an external tracking system, such as a sonar, laser, or vision system [10] and an optimal motion model can be established by continuously updating motion information through a Kalman filter with a linear distribution [11]. Moreover, an unscented Kalman filter was proposed where the current state is considered based on a Gaussian distribution, thereby allowing multi-sensor data fusion [12,13].

But in different occasions, the measurement system plays different role. Many soft or flexible sensors change the measurement system. According to the different motion capture devices can be divided into mechanical motion capture, physical inertial sensor motion capture, acoustic motion capture, electromagnetic motion capture, optical motion capture and depth camera motion capture six categories [14,15]. Besides, types of sensors based on Micro-MEMS inertial sensing technology, such as Xsens, have been developed in order to obtain high-precision results, which can be applied to motion capture system [16]. Also, plenty of attempts for applying soft or flexible sensors to motion detection or monitoring system has been made, like a sensing system capable for monitoring human body [17] and soft sensors that can monitor the movement of the wearer and robot outside the lab [18].

The measurement system developed in the present study included an inertial measurement unit system for measuring human gait movement data [19,20,21] and a visual measurement unit system for acquiring real-time walking gestures from video image sequences. The inertial measurement unit system can obtain motion inertia parameters during walking, including from the hip joint and knee joint, thereby determining the movement posture and kinematics equation. The visual measurement unit system extracts and tracks feature point sets in the environment with a single camera and then calculates the position and pose of the robot with the measurement model and by extended Kalman filtering. The two methods for gesture data acquisition are supplementary and they can improve the reliability of the detection system. The final information obtained by data fusion [22,23] is sent to the robot information processing center of the lower extremity exoskeleton via wireless transmission to provide an experimental platform and theoretical foundation for intelligent walking and feedback control for the lower extremity exoskeleton robot, so the human motion can be measured in real time and the corresponding feedback control can be facilitated. The detection system is also based on our previous experiments where we aimed to improve the comfort and safety for users of our rehabilitation training exoskeleton [24].

In our experiment condition, we included the whole walking phase in our examination as mentioned. Because VMU system and IMU system can compensate for each other, the fusing results is applicable to all walking phases, including the even stance phase. Theoretically, the detecting method can be implemented without distance or velocity limit, however, thanks to the vision limit and potential dislocation of IMU, the perfect application condition is limited to a stride frequency range from 0.5 m/s to 1.0 m/s. According to existing research, such range is suitable for test subject, who are mostly slowly-walking stroke patients.

## 2. Methods

### 2.1. Inertial Measurement Unit (IMU)

#### 2.1.1. Overview

The human body is a free system and many parameters are needed to describe a person’s walking posture accurately, such as the stride, pace, angle between the thigh and calf, angular speed and angular acceleration. However, when applying an exoskeleton robot, describing human postures becomes a constrained problem. Thus, compared with the free state of a human, the degrees of freedom (DOF) are greatly reduced for the body system and the number of corresponding parameters decreases significantly. Considering the safety of one-way walking, we are highly concerned about the joint angle because when the joint angle exceeds the normal range, the joint will appear to be unwell or damaged.

**Figure 1:**
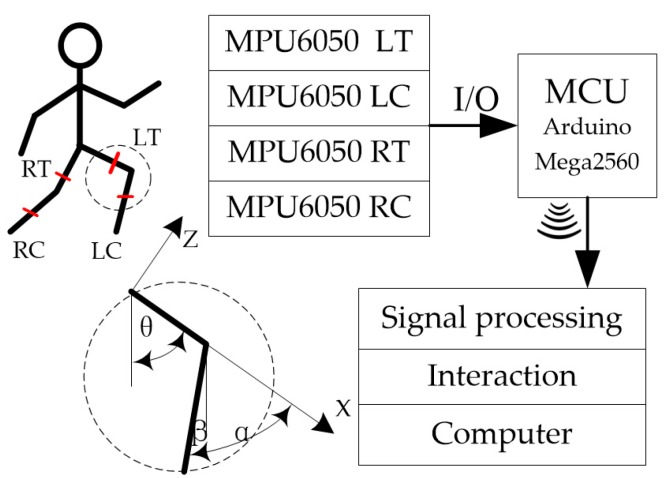
Joint angle schematic diagram of the unit employed (R: right, L: left, T: thigh and C: calf).

Considering the problems defined above, we took the knee angle α and the hip angle θ in sagittal plan as the posture measurement parameters, where the knee angle was the relative angle between the calf and thigh and the hip angle was the angle between the thigh and vertical direction, as shown in Figure 1. While functioning, the torso of test subject is fixed to exoskeleton by blinding to ensure vertical to the ground in the walking process. The ankle joints are under continuous passive motion. By combining the swing of the thigh and the calf when a person is walking, we took the anterior thigh and posterior calf as the knee angle and the positive direction of the ankle joint.

We used two attitude sensors to obtain the actual measurements, where they could measure the angle of the thigh θ and the angle of the calf β. According to the geometrical relationship in Figure 1, the relationship between the output value and the final output value of the sensor was as follows:

(1)  α=β+θ 

The final output value corresponded to the motor angle and we used this angle as feedback to control the movement of the exoskeleton robot.

In our experiment, we employed a six-DOF angle sensor module, which generated the triaxial acceleration and triaxial angular velocity as outputs. The four modules were fixed on the rehabilitation patient’s thigh and calf and thus the module moved forward. The corresponding machine coordinate system used by the module was synchronized with the human leg while the person was walking. The measurement data comprised the angle information for the human joint and the target angle could be obtained using a suitable model.

A rehabilitation patient with hemiplegia walks very slowly so the additional acceleration could be neglected in attitude calculations. We utilized the decomposition of the acceleration due to gravity to deduce the joint angle. In order to reduce the error and to obtain the best estimate of the angle, we used the angular velocity integral algorithm to solve the corresponding angle and a Kalman filter to fuse the two sets of data.

#### 2.1.2. Algorithm

Data fusion was necessary to obtain the angle between the leg and the vertical downward direction. The traditional attitude solution method for IMUs employed the Kalman filter algorithm for noise reduction with a combination of triaxial acceleration and triaxial angular velocity. We modified this fusion algorithm by substituting the decomposition of gravity for the visual identity.

First, we presented the algorithm for decomposing the acceleration due to gravity. The additional acceleration generated during walking was ignored and the triaxial acceleration measured by the sensor could be considered as the component of the gravitational acceleration on three axes. If we consider the solution for the hip angle θ as an example, then the accelerations on the three axes were accX, accY and accZ. According to vector decomposition, Equation (2) could be obtained:

(2) $$\begin{array}{c} \;\theta =\mathrm{actg}\left(-\frac{\mathrm{accZ}}{{\left({\mathrm{accX}}^{2}+{\mathrm{accY}}^{2}\right)}^{\frac{1}{2}}}\right)\; \end{array}$$

Next, we discuss the angle integral algorithm. If we set the three-axis angular velocities as groX, groY and groZ and considered the production of θ as the rotation of the x axis around the y axis, then Equation (3) can be obtained:

(3) $$\begin{array}{c} \bar{\theta }={\bar{\theta }}_{0}+\int_{t_{0}}^{t}\dot{\bar{\theta }}\mathrm{dt}\; \end{array}$$

If we select Δt as the sampling interval and the initial angle is ${\bar{\theta }}_{0}$ = 0, then the integrals defined above can be discretized as Equation (4).

(4) $$\begin{array}{c} \;\bar{\theta }\left(t\right)=\;\bar{\theta }\left(t-1\right)+\mathrm{groY}\left(t-1\right)\times \Delta t\; \end{array}$$

Next, we explained the implementation of the Kalman filter. Two sets of angular sequences could be obtained using the two algorithms defined above $\theta =\left\{\theta_{1},{\;\theta }_{2},\ldots \ldots ,\theta_{n}\right\}\;$ and $\bar{\theta }=\left\{\bar{\theta_{1}},\;\bar{\theta_{2}},\ldots \ldots ,\bar{\theta_{n}}\right\}$, where $\theta_{i}$ and $\bar{\theta_{i}}$ are the angular values measured at $t_{i}$ respectively. A state space model of the angle measurement was established to implement the Kalman filter process for the angle. The state vector is $x={\left[\theta ,\;\dot{\theta }\right]}^{T}$, the output z = θ and the discrete state mode can be obtained as:

(5) $$\begin{array}{c} \{\begin{array}{c} x\left(t+1\right)=Ax\left(t\right)+\mathrm{Bu}\left(t\right)\\ z\left(t\right)=\mathrm{Hx}\left(t\right) \end{array} \end{array}\;$$

where $A=\left(\begin{array}{cc} 1 & \Delta t\\ 0 & 1 \end{array}\right)$, $B=\left(\begin{array}{c} \frac{{\left(\Delta t\right)}^{2}}{2}\\ \Delta t \end{array}\right)$, u(t)=θ(t) and H=(1,0).

According to the model above, five iterative formulae could be obtained for the Kalman filter:

(6) $$\begin{array}{c} \{\begin{array}{c} {\hat{x}}^{-}\left(t\right)=A\hat{x}\left(t-1\right)\\ P^{-}\left(t\right)=\mathrm{AP}\left(t-1\right)A^{T}+Q\\ K\left(t\right)=P^{-}\left(t\right)H^{T}{\left({\mathrm{HP}}^{-}\left(t\right)H^{T}+R\right)}^{-1}\\ \hat{x}\left(t\right)={\hat{x}}^{-}\left(t\right)+K\left(t\right)\left(z\left(t\right)-{H\hat{x}}^{-}\left(t\right)\right)\\ P\left(t\right)=\left(I-K\left(t\right)H\right)P^{-}\left(t\right) \end{array} \end{array}.\;$$

where P(t). is the covariance matrix, K(t) . is the Kalman gain matrix and Q is the covariance error matrix. If we select $P\left(0\right)=\left(\begin{array}{cc} 1 & 0\\ 0 & 1 \end{array}\right)$, we could find the appropriate $Q=\left(\begin{array}{cc} 10 & 0\\ 0 & 0.003 \end{array}\right)$. and replace $z\left(t\right)-{H\hat{x}}^{-}\left(t\right)$ with $\theta \left(t\right)-\;\bar{\theta }\left(t\right)$. Thus, the optimal estimated value $\bar{\theta }$ of θ could be obtained using the iterative formulae and the optimal estimates of the remaining joint angles could also be generated in the same manner.

#### 2.1.3. Hardware Implementation

We utilized a Kalman filter for data fusion in order to obtain the best estimates of the angle measurements. After evaluating the sensor measurement results, we obtained the results shown in Table 1. The actual angles were measured using a protractor. In order to consider the protractor measurement error and the floating position of the module, we determined the mean values based on 50 measurements of the angle values, which were closest to the actual angles. As shown in Table 1, when the actual angle was close to 0° or 90°, the measurement error was relatively large but the average error was within 1% and thus the error was in an acceptable range.

We employed four MPU6050 angle sensors to measure the angles of four joints and the data were acquired by an Arduino Mega2560. The angular velocity measurements, triaxial acceleration and sampling time were transmitted via Bluetooth. We used a robot operating system (ROS) to run a node for each angle, as well as for processing the received data, conducting filtering, storing data, printing out the results and other processes. The MPU6050 module communicated with the microcontroller via IIC (Inter-Integrated Circuit) communication, where each module had two register addresses, that is, 0 × 68 and 0 × 69 and the last digit was controlled by the AD0 pin on the module. We employed the following methods in order to use one Arduino board to receive the data from the four modules. Four AD0 pins were set at high levels before reading the 0 × 69 data, so the sampling time interval for each module was four times the cycle program running time. In the communication mode, we employed IIC communication between the four MPU6050 modules and the Arduino board. Signals were transferred between the Arduino board and computer via Bluetooth, as shown in Figure 1, which was convenient and quick. The data were processed in the computer to reduce the operating burden on the Arduino MCU (Micro Controller Unit) by eliminating the Kalman filter program. Thus, the cycle time was shortened for the Arduino program, thereby increasing the sampling frequency so more data were obtained within a specific time.

### 2.2. Visual Measurement Unit (VMU)

#### 2.2.1. Overview

In wearable networks, the data sent back by sensors can cause certain transitions due to environmental or hardware problems. The aim of visual environment monitoring was to collect and process optical information, to obtain the data and to merge and supplement the data sent back by the wearable sensor, thereby improving the accuracy of signal extraction.

**Figure 2:**
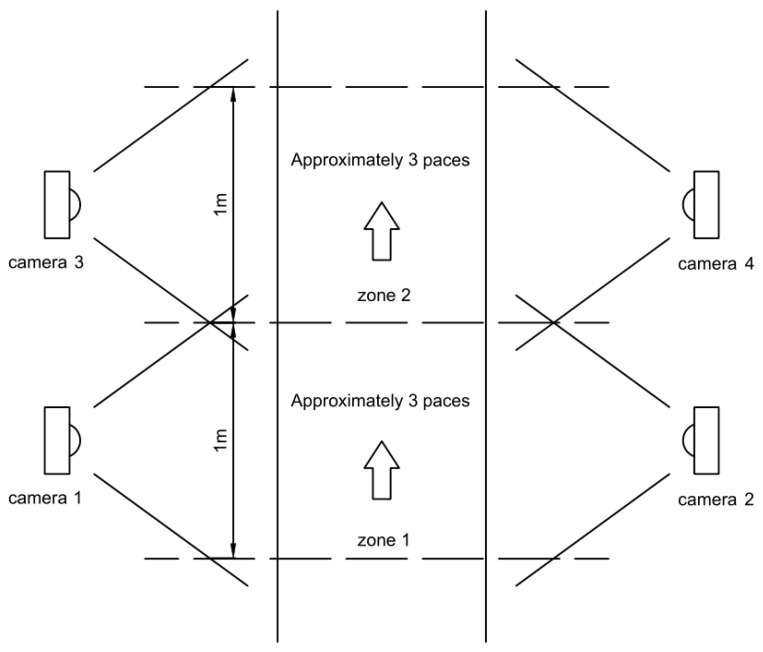
Layout diagram of workplace for exoskeleton.

The patient walks in an exoskeleton with small stride, low stride frequency and straight-line walking. In the designed rehabilitation program, there are a group of cameras distributed on both sides of the path, thus the whole gait circle of the patient can be captured while walking. According to the current position of the patient in the path, certain cameras are activated to work, as the following Figure 2 shows.

The figure shows the program environment with the path zone separated by dotted lines. Each side of a zone part is placed a camera. All the cameras are connected to a central controller (PC) and keep running and the controller determines which cameras to be activated. In particular, when the patient is in zone 1, camera 1 and 2 detect the paces of the two legs; and when the patient walks cross the dotted line and be in zone 2, camera 3 and 4 are ready to work. The instant when camera 3 and 4 detect the leg across the dotted line triggers the switch from camera 1 and 2 to camera 3 and 4 in the main data process module as its data sources. There is horizon margin for each camera focusing on each zone to ensure the source switch being smooth. This process environment can be extended for a longer path. In the experiment, in the general, patient walks within the range of camera 1 and 2.

**Figure 3:**
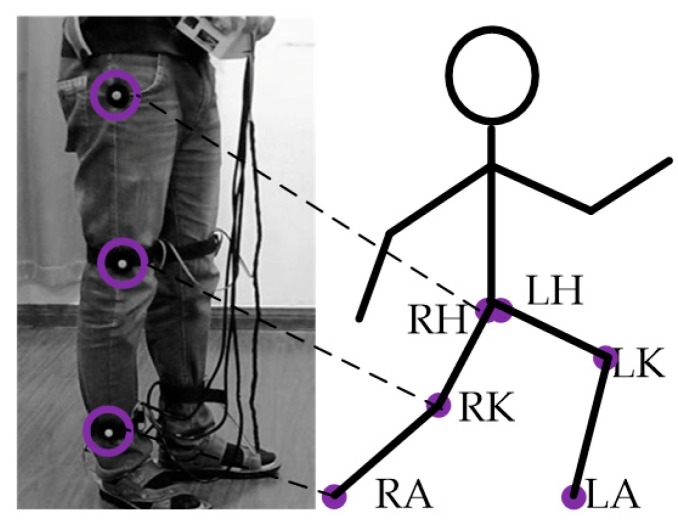
Marker points employs (R: right, L: left, H: hip joint, K: knee joint and A: ankle joint).

The key aim of capturing gait information under optical conditions was to correctly detect and locate a specific mark in the video stream and to record it in real time. In our experiment, special markers were pasted on the hip, knee joint and ankle joint, which were the key points for obtaining gait information. After obtaining the real-time coordinates of these three key points (Figure 3), we could determine the swing angles of the lower limbs and their variations with time during walking.

**Figure 4:**
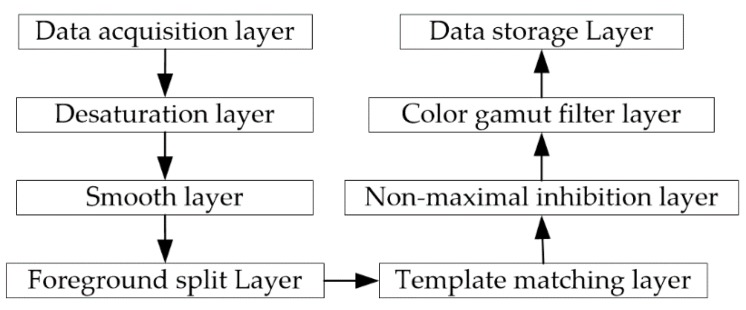
Hierarchical model of visual environment monitoring.

In order to structure data collection and processing, as well as to enhance degree of modularity and scalability, the overall logic process of visual environment monitoring was organized into a hierarchical stack. Each layer was built on top of the previous layer and the data were subjected to a specific operation in each layer, before they were transmitted to the next level, as shown in Figure 4.

#### 2.2.2. Architecture Implementation

(1) Data acquisition layer

The function of the data acquisition layer was to ensure the successful input of optical information and the transmission of original data. In practice, the optical information was captured in the form of data frames and the video stream was iterated rapidly between the data frames, which included the information needed to detect the gait.

In the computer, the data frames captured by the camera were in the form of a matrix array, so the data extraction process obtained the coordinate positions of the marked point in each frame by mathematical processing and conversion of the matrix array. The data acquisition layer was not responsible for extraction but instead it invoked the camera and connects the camera data flow with the data extraction program. The data frames corresponding to the matrix array were passed continuously up the hierarchical structure of the model.

In general, by default, the data captured by a camera were represented in the BGR color space format. BGR had three channels for the three matrices in the array representing the blue, green and red parts components. Each pixel color could be divided into a mixture of these three colors in different proportions. After being divided, the proportions of each color were preserved in the corresponding position of each channel. In order to retain most of the original information, instead of processing the matrix array, the data acquisition layer passed it directly to the upper model.

(2) Desaturation layer

In the BGR format, the image data retained most of the optical information but not every operation required all of the information when searching for the marker points. Thus, the useful information could be represented better by compressing a three-channel matrix array into a single channel in order to speed up the search for markers, although some of the data would be lost, where a color image was compressed into a grayscale image. Matrix conversion was performed as follows:

(7)  Y=0.114×B+0.587×G+0.299×R 

where Y is the matrix representing the grayscale image and a larger number indicates that the pixel representing this position is whiter, whereas a lower number denotes a darker pixel. The desaturation layer sends the BGR image and the processed gray image to the upper layer. The upper level could select and process these two types of data according to the necessary requirements.

(3) Smooth layer

Each input frame generated noise due to natural vibrations, changes in illumination, or hardware problems. The smooth processing of the data frame could effectively remove the noise and avoid interference from the detection process.

Applying Gaussian blur to process each pixel in an image was an effective method for smoothing. For any pixel point, Gaussian blur took the weighted average of all the surrounding pixels, where the weight was distributed according to a normal distribution. The weight was larger when the points were closer and smaller when the distance between the points was greater. In practice, we found that taking the target pixel as the center and employing a 21 × 21 matrix provided the best performance when using the weighted average of the surrounding pixels in the matrix to remove the noise.

The desaturation layer and smoothing layer could be employed as a pretreatment layer, which was convenient for data processing, as described in the following.

(4) Foreground split layer

In the real world, testers will walk through the lens of a monocular camera from left to right and expose the marks on their sides to the camera during the process. When capturing optical information using a monocular camera, the three-dimensional environment was mapped onto a two-dimensional plane. From the perspective of the two-dimensional video, the camera was still so the whole video could easily be divided into the foreground and background, where the foreground was the area where the movement of the tester was located. The tester walked into the frame from one end of the lens and left from the other end, so the foreground area also slid continuously in the video. Excluding the testers, the background comprised the areas that filled the whole environment, that is, parts other than the foreground area. These areas were characterized as remaining stationary in the entire video and they were obscured by the movements in the foreground area. The foreground area occupied a smaller area of the whole video frame, whereas the background area occupied a larger area. Clearly, the area where the target marker was located must be in the foreground. Scanning the background area would consume power and waste time but it would also lead to incorrect results when the matching threshold setting was low and it could further disturb the extraction of marker point data. If the foreground region could be split from the entire data frame, then the search would only focus on this area and the search efficiency could be improved greatly, regardless of the large background area.

The foreground region was divided from the data frame based on the foreground segmentation layer. In actual situations, at the beginning of the video test, the testers had not yet entered the shot and all of the image components in the first frame of the video stream belonged to the background map region and thus this frame was designated as the reference background frame, which was taken as the basis for dividing the foreground and background in the subsequent data frames.

The basis of foreground division involved binarization of the threshold value. First, for any data frame M, the calculation should conform to the following rules:

(8) $$\;\mathrm{absdiff}\left(I\right)=\left|M\left(I\right)-M_{o}\left(I\right)\right|\;$$

where $M_{o}$ is a reference background frame in video stream, I represents a specific position in the data frame and absdiff is a matrix array.

In a grayscale image, the values of various elements ranged from 0–255 and the values of the various elements of absdiff were within 0–255. Absdiff could also be regarded as a grayscale image. We set the thresholds and conduct binarization to transform absdiff into complete black and white images, where the white areas and black areas roughly represented the foreground and background distributions, respectively. Inevitably, large amounts of noises were present in the images after binarization because data points were compared to various threshold value at the junctions of the foreground and background values. It was difficult to divide the ideal black and white demarcation line. Expansion could be used to trim burrs and eliminate noise points. First, we defined a structure matrix of 5 × 5 and the rules for generating the structure matrix were as follows:

(9) $${\;E}_{\mathrm{ij}}=\{\begin{array}{c} 1,\;\mathrm{if}\;\sqrt{{\left(i-3\right)}^{2}+{\left(j-3\right)}^{2}}<\sqrt{5}\\ 0,\;\mathrm{otherwise} \end{array}\;$$

The structure matrix size could be adjusted according to the actual situation and $\sqrt{5}$ in the generating rules should be changed to the appropriate number. After the structure element had been generated, it could be used to traverse the image and an expanded binary image could be obtained after processing according to the following rules:

(10) $$\;\mathrm{dilate}\left(x,y\right)=\max\;\mathrm{absdiff}\left(x+x^{\prime },y+Y^{\prime }\right),\;E\left(x^{\prime },\;Y^{\prime }\right)\neq 0\;$$

**Figure 5:**
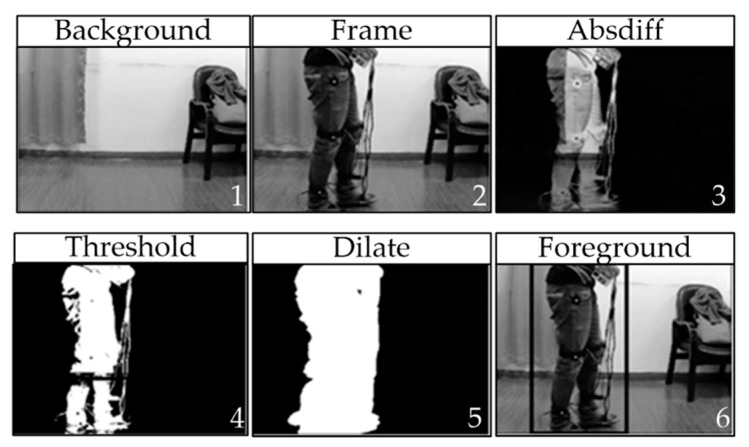
Foreground segmentation process.

In the dilate image, the white part was expanded to a contiguous area and the boundary was more obvious. Finally, according to the distribution of the white part, a complete area was formed in the data frame with a rectangle, which was taken as the foreground region and the remaining area was the background area, as shown in Figure 5.

The foreground segmentation layer passed the data frames in the two-color formats to the upper level but it also provided the rectangular coordinate information to the upper foreground area, so the upper layer could focus on the foreground area.

(1) Template matching layer

The template matching layer provided the core function of the program, where it filtered and extracts the marker points from the image information and recorded their coordinates. Before processing, the template-matching layer was preloaded with the three marker point templates, which then traversed the foreground area as a benchmark. When the template traversed to a region where the foreground (x, y) was at the center, the following formula could be applied to obtain the normalized difference squared sum:

(11) $$\;R\left(x,y\right)=\frac{\sum_{x^{\prime },y^{\prime }}{\left(T\left(x^{\prime },y^{\prime }\right)-I\left(x+x^{\prime },y+y^{\prime }\right)\right)}^{2}}{\sqrt{\sum_{x^{\prime },y^{\prime }}T{\left(x^{\prime },y^{\prime }\right)}^{2}\cdot \sum_{x^{\prime },y^{\prime }}I{\left(x+x^{\prime },y+y^{\prime }\right)}^{2}}}$$

where R was regarded as a variable related to the relative degree and the matching degree of the corresponding pixel was better for the surrounding area and the template when the value of R was smaller.

**Figure 6:**
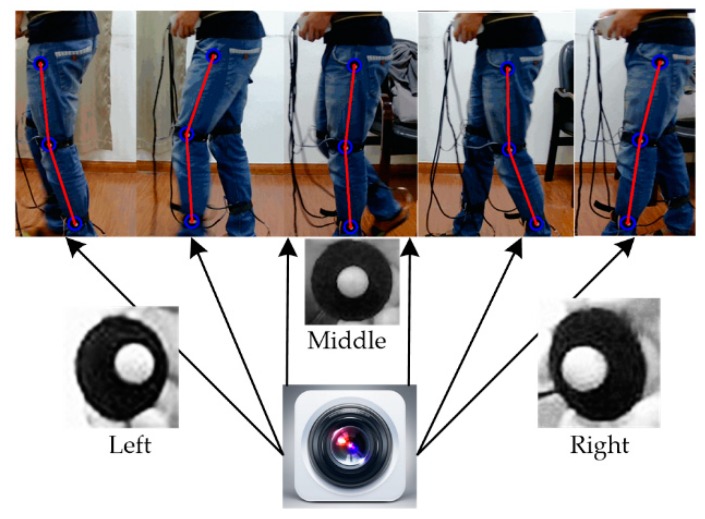
Different projection patterns for marker points.

In practice, the marker points on the tester would not always be facing directly at the camera, so the marker point image captured by the camera may be an irregular oval state rather than a circle, as shown in Figure 6. In addition to the markers facing the camera directly, they may be tilted to the left or right and three templates were designed for these three cases. Thus, the matching degree for each of the three templates and the region were calculated as the frame was traversed and the lowest result was maintained, as follows.

(12) $$\;R\left(x,y\right)=\min\frac{\sum_{x_{i}^{\prime },y_{i}^{\prime }}{\left(T\left(x_{i}^{\prime },y_{i}^{\prime }\right)-I\left(x+x_{i}^{\prime },y+y_{i}^{\prime }\right)\right)}^{2}}{\sqrt{\sum_{x_{i}^{\prime },y_{i}^{\prime }}T{\left(x_{i}^{\prime },y_{i}^{\prime }\right)}^{2}\cdot \sum_{x_{i}^{\prime },y_{i}^{\prime }}I{\left(x+x_{i}^{\prime },y+y_{i}^{\prime }\right)}^{2}}},\;i=1,2,3\;$$

After traversing the image, the R values corresponding to most of the pixel points were fairly large, which indicated that these regions did not match the template, whereas the R values for some pixels were in a very small range, thereby indicating that this area was close to the template. If we took 0.1 as a threshold, the pixel areas with R values greater than this value were discarded, whereas the center pixel coordinate data were recorded for the region where the marker point was considered to be located. Finally, the template matching layer passed the image data and the marker point coordinated to the upper layer for further processing.

(2) Non-maximal inhibition layer

When we checked the coordinate data obtained by the template matching layer, there was obvious overlapping where the points with similarly good matching degrees often appeared together. Their difference squared sums were all less than the threshold value. Therefore, they were recorded but the matching windows corresponding to these points obviously overlapped with each other. The center of the overlapping area was generally a marker point. This cluster of coordinates all represented the same coordinates, so the best representative was selected from the cluster to denote the marker point and the remaining coordinates were discarded as noise:

(13) $$\left(x,y\right)=\min\;R\left(x^{\prime },y^{\prime }\right),\;\left(x^{\prime },y^{\prime }\right)\in \mathrm{Range}\;$$

where Range was the region where a cluster of coordinates was located and the coordinate with the lowest R-value was selected as the best result in terms of the matching degree for the coordinates of the cluster at a certain position. The non-maximal inhibition layer passed the filtered coordinate points and image data to the upper layer.

(3) Color gamut filter layer

The coordinate points obtained at this point comprised the locations of each marker point in theory but the template matching layer was not excluded because of the changes in other areas in the foreground. The foreground area was changing constantly and an incorrectly identified area did not continue to cause interference, so the error was random and uninterrupted. Thus, color gamut filtering was employed as a simple additional discriminant rule for each target area to eliminate this interference.

Color gamut filtering employs color images that have not been previously used for recognizing mark points. The previous screening step was based on the grayscale image and thus the color information in the color images was not used, thereby increasing the computational efficiency. In the color gamut filter layer, this part of the information was employed based on comparisons of the colors of the coordinate points and the recognition results were optimized further.

First, color gamut filtering converted an image in BGR format to HSV (hue (H), saturation (S) and lightness (V)) format using the following formulae:

(14)  V=max(R, G, B) 

(15) $$\;S=\{\begin{array}{c} \frac{V-\min\left(R,G,B\right)}{V},\;V\neq 0\\ 0,\;V=0 \end{array}$$

(16) $$H=\{\begin{array}{c} 60\cdot \frac{G-B}{V-\min\left(R,G,B\right)},\;V=R\\ 120+60\cdot \frac{B-R}{V-\min\left(R,G,B\right)},\;V=G\\ 240+60\cdot \frac{R-G}{V-\min\left(R,G,B\right)},\;V=B \end{array}\;$$

In the HSV color space, three parameters are more suitable for comparing the degree when approximating two different colors than those in the BGR space. In order for a point to be measured, it passed the test if its core color was white; otherwise, it could not pass the test and the coordinates would be discarded. We determined whether a color belonged to the white range based on the S and V parameters. If the following conditions were satisfied, then the point was white:

(17)  0≤S≤0.5 

(18)  0.5≤V≤1 

This method could be used as an auxiliary means to quickly filter the locations of marker points. The data were filtered relatively well in the previous layers, so the accuracy requirement was not very demanding. The non-maximal inhibition layer and gamut filter layer aimed to further improve the matching coordinates, so they could be collectively referred to as the post-processing layer.

(4) Data storage layer

In addition to the data acquisition layer, the data storage process aimed to ensure that the data passed to this layer were preserved correctly. This data layer contained a color picture and the grayscale image corresponding to the array matrix. A number of filters were applied to determine the final locations of the marker point coordinates. In the video shooting process, the incoming data stream was quickly and effectively stored in different locations in the data storage layer, where the picture was stored in a video frames format and the coordinate information was written in chronological order. If the other layers were interrupted for various reasons during the transmission process, the data storage layer must be kept waiting and it could not be disconnected. It was not possible to overwrite previous data in multiple storage tasks.

### 2.3. Data Fusion Unit

#### 2.3.1. Overview

The main idea of the facilities is to find a proper way to support the measurement of the action of patients in rehabilitation exoskeleton, which focuses on a kind of motion with both small stride and frequency. Commonly used sensors include inertial sensors and optical sensors. Inertial sensors provide high accuracy and are prone to generate integral drift. Optical sensors are generally less accurate. Under this situation, the fusion of data from wearable sensors and optical sensor can bring the motion information with a better accuracy performance.

Compared with any individual data source, multiple data sources could provide more accurate measurement information [25,26]. For vision processing information, the sampling period was limited by the calculation speed and visual acquisition was susceptible to the background and brightness, which decreased the credibility of vision data. The output signal contained a large amount of white Gaussian noise for information from the IMU. Due to the acceleration of the legs, the vector composition of triaxial acceleration could not indicate the global direction of gravity precisely, so the acceleration direction data could not be used as the reference for gravity.

#### 2.3.2. Fusion Algorithm

Basically, we applied low-level data fusion to combine several sources of raw data to produce new raw data, where it was expected that the fused data would be more informative and synthetic than the original inputs. The precision of inertial sensor is higher when the speed is stable but the error will be larger when the velocity changes and the precision of the vision sensor is lower than the displacement sensor when the speed is stable but the tracking performance is better when the velocity changes. Therefore, based on the Kalman estimator, the fusion strategy of different stages and weights is adopted to fuse the two groups of data.

The steps of the Kalman estimator are as follows: Firstly, Setting the initial value of X and P. X is the state of the system at each moment. P is the covariance of the error of the measurement value. Secondly, enter the iteration and simplify Equation (6) into three iterations:

(19) $$\;K\left(t\right)=P^{-}\left(t\right)H^{T}{\left({\mathrm{HP}}^{-}\left(t\right)H^{T}+R\right)}^{-1}$$

(20) $$\hat{x}\left(t\right)=x\left(t-1\right)+K\left(t\right)\left(z\left(t\right)-\mathrm{Hx}\left(t-1\right)\right)$$

(21) $$\;P^{-}\left(t+1\right)=\left(I-K\left(t\right)H\right)P^{-}\left(t\right)+Q\;$$

On this basis, the algorithm is further optimized. z(t) in the above formula is the observed value of step t. In the experiment, there are two groups of sensors, so we have two observations. In the phase of steady velocity, a relatively large weight is given to the observation value of the displacement sensor. A relatively large weight is given to the visual sensor during the period of speed change. Calculate the weighted average of the two observations. Weight is very important. We can get the optimal weight of measurement system through the optimally weighted fusion calculation of minimum variance and the fitting of multiple sets of data.

Suppose N sensors are used to observe an unknown quantity Z. Observations values of the sensor are $\left\{Z_{j}\right\}\left(j=1,2,\ldots ,N\right)$, respectively.

The observation of the j sensor can be expressed as

(22) $$\;Z_{j}\left(t\right)=Z\left(t\right)+n_{j}\left(t\right)\;$$

$n_{j}\left(t\right)\;\mathrm{is}\;\mathrm{noise}\;$. Variance of $n_{j}\left(t\right)$ is expressed as

(23) $$\;\sigma_{j}^{2}=E\left[n_{j}^{2}\left(t\right)\right]\;$$

If the observations are unbiased and independent of each other, the estimate of Z can be expressed as

(24) $$\hat{Z}=\sum_{j=1}^{N}W_{j}Z_{j}\;$$

$W_{j}$ is the weighted coefficient and $\sum_{j=1}^{N}W_{j}=1$.

The estimated variance is

(25) $$\;\sigma^{2}=\sum_{j=1}^{N}W_{j}^{2}\sigma_{j}^{2}\;$$

$\sigma_{j}^{2}$ is the noise variance of sensor j.

In order to get $W_{j}$ to make $\sigma_{j}^{2}$ the minimum variance, we construct the auxiliary function.

(26) $$\;f\left(W_{1},W_{2},\ldots ,W_{N},\lambda \right)=\sum_{j=1}^{N}W_{j}^{2}\sigma_{j}^{2}+\lambda \left(\sum_{j=1}^{N}W_{j}-1\right)\;$$

Now, the question becomes to solve the minimum value problem when $\sum_{j=1}^{N}W_{j}=1$.

It can be solved as follows:

(27) $$\;{\;W}_{j}=\frac{1}{\sigma_{j}^{2}\sum_{i=1}^{N}\frac{1}{\sigma_{i}^{2}}},\;j=1,2,\ldots ,N\;$$

**Figure 7:**
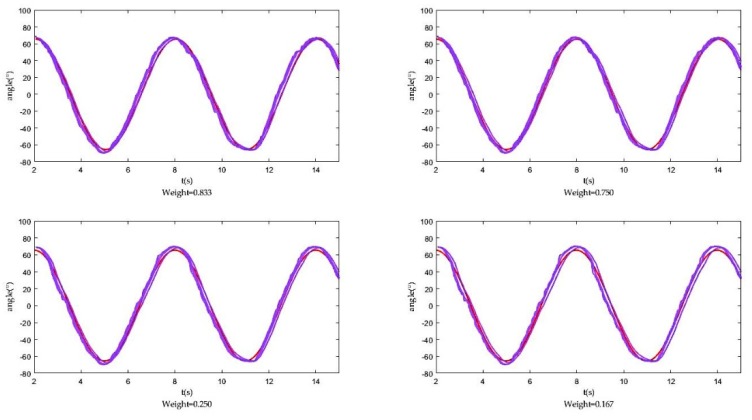
Fusion results of four groups with different weight.

From the above analysis, we can see that the optimal weighting factor $W_{j}$ is determined by the variance $\sigma_{j}^{2}$ of each sensor j but it is generally unknown. Therefore, we designed an experimental platform to test the measured values of each sensor and get this $\sigma_{j}^{2}$. Here we show the fusion result of four groups with different weight, as the following Figure 7 shows.

#### 2.3.3. Proof of Algorithm

**Figure 8:**
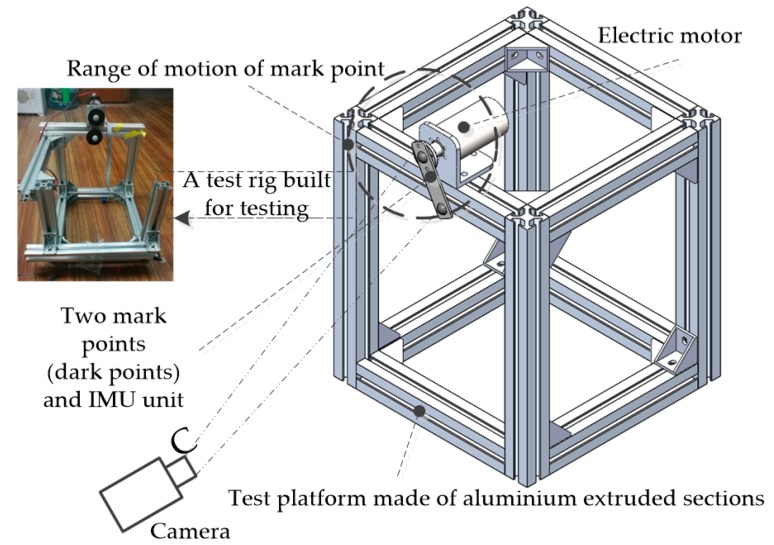
Test platform.

We obtain the weight from the test platform shown in Figure 8, where the pendulum was actuated by a stepper motor and the angle of the pendulum was controlled to profile a sinusoid.

**Figure 9:**
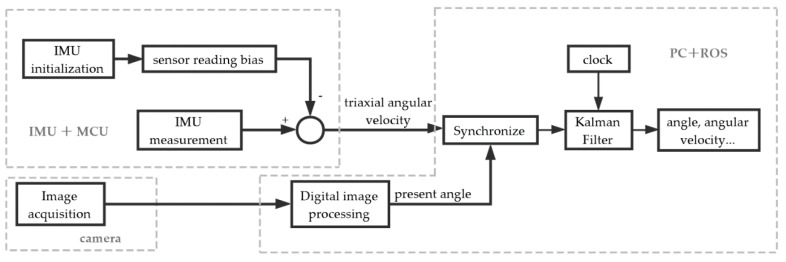
Data flow diagram.

In architecture implementation, the Arduino board obtained the sensor reading and completed the initialization of the IMU. In this process, the user should keep the inertia sensor steady until the initialization is finished so the sensor could measure the angular velocity bias caused by the temperature and measurement error and the sensor reading bias was then stored as a constant. The difference between the sensor reading and bias was transmitted to the upper computer for further calculation, as shown in Figure 9.

On the master computer, we used ROS to run different threads on different nodes, which communicated with each other via a topic method. The IMU node published attitude data continuously and the vision node published angle data continuously. These two sources of information published messages asynchronously, so the Kalman filter node could not run normally. To cope with this problem, another node was added to provide the function of a first-order hold, which could synchronize the vision node messages with the IMU node messages. All of the data were written into .txt files for subsequent processing.

**Figure 10:**
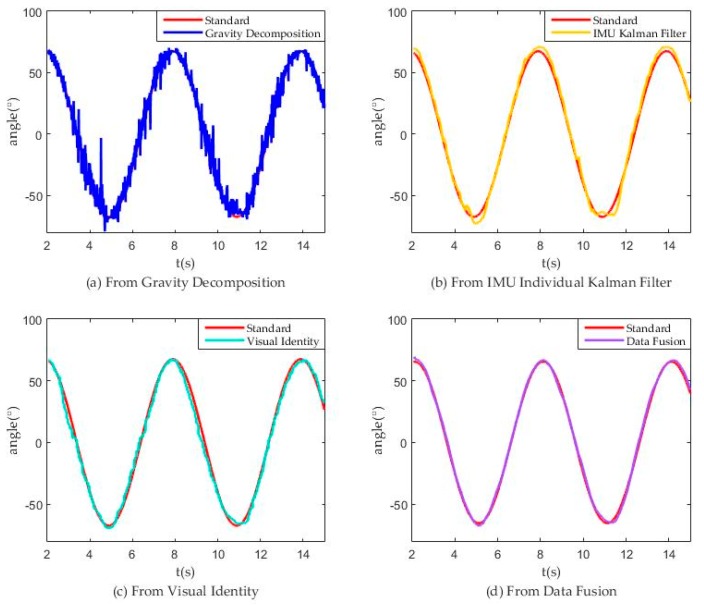
Model test data from different sources.

In the test platform, we make stepper motor run as the standard curve and obtain the following measurement curve. Figure 10a show that the noise obtained from gravity decomposition was very large, which greatly reduced the precision. Figure 10b shows that using the Kalman filter to combine the IMU triaxial acceleration and triaxial angular velocity still obtained results that were not very smooth. According to Figure 10c,d, we specified a variable E to characterize the error between the results and the ideal values:

(28) $$\sigma^{2}=E=\frac{\sum_{n=195}^{1447}{\left({\mathrm{angle}}_{n}-{\mathrm{standard}}_{n}\right)}^{2}\;}{1447-195}\;$$

Here, n = 195~1447 is corresponding to t = 2.0107 s~15.0016 s, thereby obtaining:

(29) $${\;E}_{\mathrm{vision}}=20.0531,{\;E}_{\mathrm{fusion}}=3.7433,\;{\;E}_{\mathrm{IMU}}=13.2981\;$$

(30) $$E_{\mathrm{vision}}>E_{\mathrm{IMU}}>E_{\mathrm{fusion}}$$

**Figure 11:**
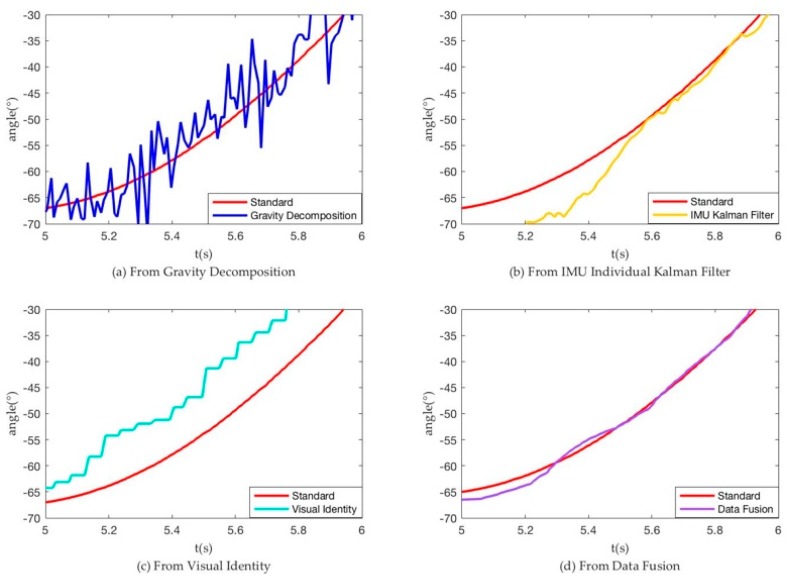
Partial enlarged drawing of Figure 10 (Between 4th and 5th second).

The value of variable E shows that the data fusion results were much better than the vision results because of less error and noise. Figure 11 shows magnification fusion result (Between 4th and 5th second).

## 3. Tests and Results

### 3.1. Exoskeleton Test without Load

**Figure 12:**
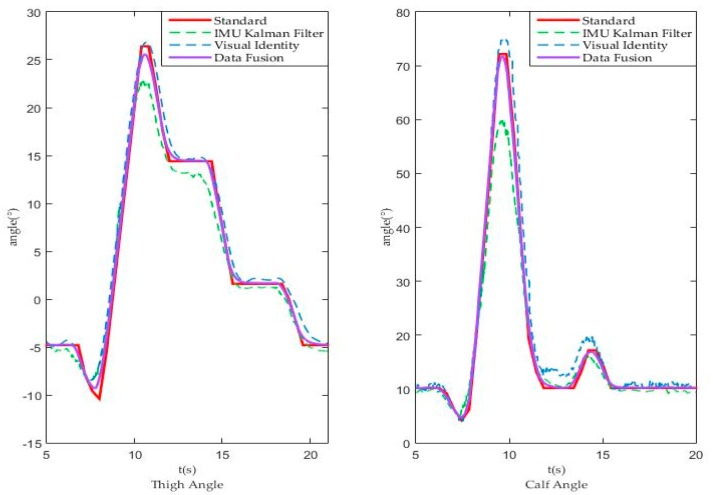
Exoskeleton test without load from different sources.

**Figure 13:**
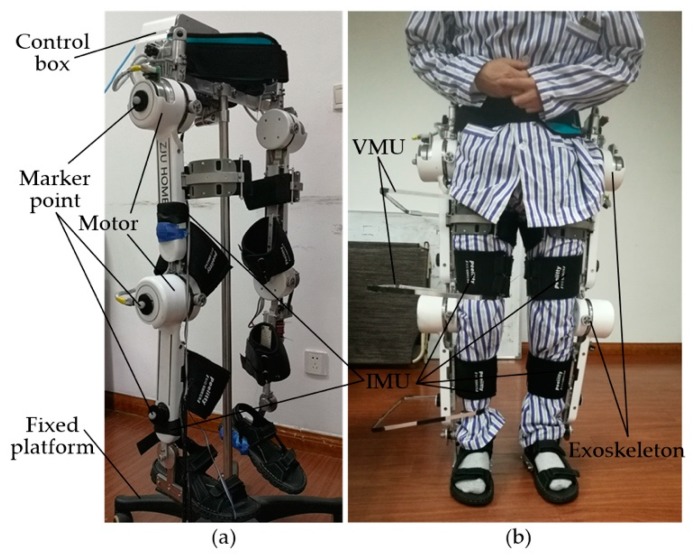
Exoskeleton without load (**a**) and human with exoskeleton (**b**).

To verify the applicability and accuracy of the weight obtained above, we used the same vision identity and IMU measurement methods to obtain the angle-time curve and to verify the advantages of the fusion process with the same weight in an exoskeleton without load. In the test, we make exoskeleton run as the standard curve and obtain the following measurement curve, as shown in Figure 12. Picture of exoskeleton testing without load can be found in Figure 13a.

The left picture shows the change of the corresponding angle of the thigh in a walking cycle, the red solid line is the standard reference curve of exoskeleton movement and the green and blue dashed lines are the IMU and visual method respectively. The purple solid line is the fusion result curve. The right image shows the change of the calf angle in a walking cycle and several curves are the same as the left.

We specified a variable E to characterize the error between the results and the ideal values:

(31) $$\;E=\frac{\sum_{n=358}^{1525}{\left({\mathrm{angle}}_{n}-{\mathrm{standard}}_{n}\right)}^{2}\;}{1525-358}\;$$

Here, n = 358~1525 is corresponding to t = 5.001 s~21.005 s, thereby obtaining:

For thigh,

(32) $$\;{\;E}_{\mathrm{vision}}=100.59,{\;E}_{\mathrm{fusion}}=27.06,\;E_{\mathrm{IMU}}=91.33\;$$

(33) $$\;E_{\mathrm{vision}}\;>E_{\mathrm{IMU}}\;>{\;E}_{\mathrm{fusion}}\;$$

The value of variable E shows that the data fusion results were much better than the vision and IMU results because of less error and noise.

For calf,

(34) $$\;{\;E}_{\mathrm{vision}}=185.01,{\;E}_{\mathrm{fusion}}=53.38,{\;E}_{\mathrm{IMU}}=218.10\;$$

(35) $$\;E_{\mathrm{IMU}}\;>E_{\mathrm{vision}}\;>{\;E}_{\mathrm{fusion}}\;$$

The value of variable E shows that the data fusion results were much better than the vision and IMU results because of less error and noise. This exoskeleton test without load results show that the weight and measurement system is suitable and available.

### 3.2. Human Test with Exoskeleton

Finally, we used this method to control the trajectory of the motors in an exoskeleton system, as shown in Figure 13b. During the period of walking, the actual joint trajectories of the volunteer patient (he has agreed to cooperate with us) can be deviated from the standard trajectories, due to the non-rigid connection between exoskeleton and human body. The angle values of thigh and knee joints are taken to be the feedback quantities to decrease the difference between standard and experiment trajectories.

Here, we need to make one point. Considering the data jitter which is introduced to the measurement system by non-negligible vibration of the patient when applying human test, several effective measures are taken to keep the vibration error limited and in a controllable range. Specifically, low inertia material is chosen to make the support frame with an arm length of no more than 250 mm, which ensures a relatively limited vibration in an application scenario with small stride and frequency. Furthermore, we apply the debounce algorithm to eliminate the detected vibration. Thus, with non-negligible vibration occurring in the process, acceptable visual information can be obtained.

**Figure 14:**
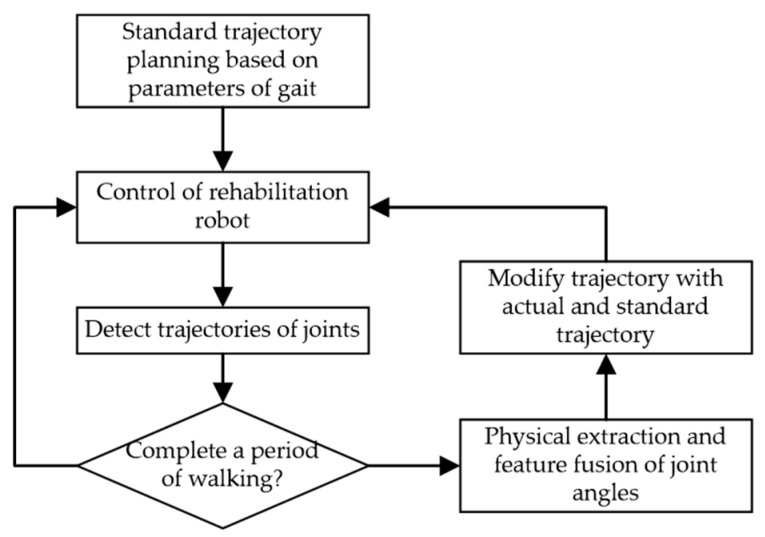
Actuate Curve Generation Process.

The whole human test with the exoskeleton is shown in Figure 14.

Though the curve fitting, the standard trajectory can be expressed as $M_{0}$. The function of standard trajectory is as follows:

(36) $$\;F_{0}\left(x\right)=\sum_{i=0}^{n}M_{0}\cos\left(\mathrm{it}\right)$$

For the kth period of walking, the modified control trajectory is represented by M(k) and the actual experiment trajectory is represented by $\tilde{M}\left(k\right)$. Considering the human-exoskeleton system as a transformation system, M(k) and $\tilde{M}\left(k\right)$ are the input and output of it. To decrease the error between the actual experiment trajectories and the standard trajectories, we describe it:

(37) $$\;\tilde{M_{i}}\left(k\right)-M_{i}\left(k\right)\to 0\;$$

(38) $$\tilde{M}\left(k\right)-M_{0}\to 0\;$$

With the Discrete Fourier Transform (DFT), the actual experiment trajectory of the kth period of walking can be changed in a similar way. Based on Newton’s string-cross-method, the iteration process can be summarized as:

(39) $$\;M_{i}\left(k+1\right)=M_{i}\left(k\right)-\frac{\tilde{M_{i}}\left(k\right)-M_{0}\left(k\right)}{\tilde{M_{i}}\left(k\right)-\tilde{M_{i}}\left(k-1\right)}(M_{i}\left(k\right)-M_{i}\left(k-1)\right)\;$$

**Figure 15:**
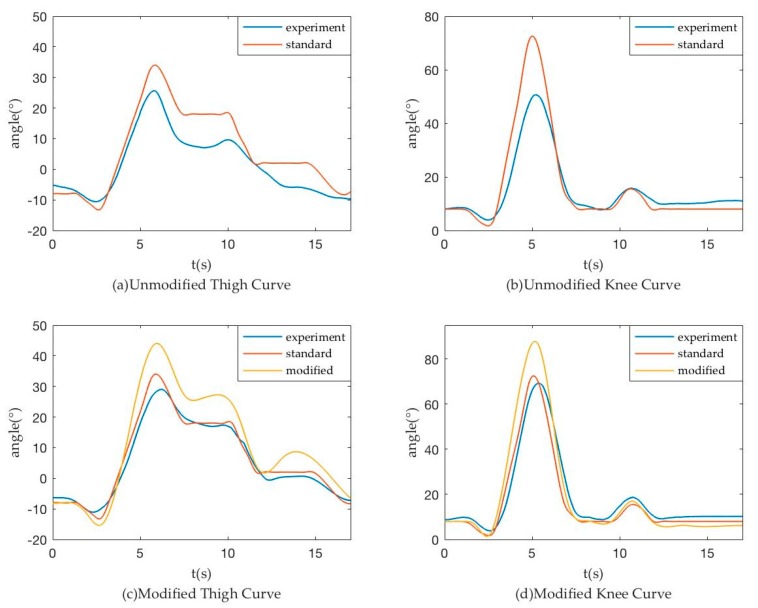
Modified Results.

After several steps of iterations, we can obtain an appropriate actuate curve to make the human body experiment curve approximate the standard curve. In the experiment, the full system was divided into several periods. During the first period of walking, the control track is original and the actual experiment trajectories of joints were taken by measurement system. Since the next period, the actual trajectories of previous period were used as the feedback information to control the walking gait of patients. Our method played an effective effect in the whole system, as shown in Figure 15. It provides a set of accurate real-time measurements for patients in rehabilitation and data support for effective control of exoskeleton robot.

## 4. Discussion and Future Study

Overall, we found that this measurement system for patients in a rehabilitation exoskeleton robot is effective. The measurement system can provide accurate real-time input for the feedback control of exoskeleton and we have verified it in our experiment. The measurement system is used for rehabilitation exoskeleton robot worn by slowly-walking patients and costs lower. However, the current measurement system is still unsatisfactory and can be improved further in the future study.

We only considered the joint angle in the sagittal plane of a human body and omitted the other two angles for this exoskeleton robot. Measurement of joint angle in the coronal and horizontal planes can be achieved in the next generation of intelligent rehabilitation exoskeleton robot, thereby providing a more accurate description of the body posture.

The current measurement system is required for use in more complex environments. The template recognition method could scan the video frames rapidly and identify locations of markers. When the marker changes to some extent, identification will fail. Given the presence of redundant noise and errors, to prune the data further is necessary. To improve the performance, more template sizes can be introduced and a gradient with different sizes can be explored further.

## Figures and Tables

**Table 1:** Measurement accuracy of detection using MPU6050 angle sensors.

Practical Angle (°)	Measurement Angle (°)	Relative Error (%)
0	0.69	/
15	15.44	2.93
30	30.24	0.8
45	45.12	0.27
60	60.12	0.2
75	75.08	0.11
90	89.65	0.39
